# Supplementary material for: GmUFO1 Regulates Floral Organ Number and Shape in Soybean
Source: Int J Mol Sci. 2023 Jun 2;24(11):9662. doi: 10.3390/ijms24119662 (PMC10253600; doi:10.3390/ijms24119662)

Supplementary Data

Table S1. Primers used in this paper.

Primer ID	Sequence	Note
OL13180	ATGGAAGGTTTTCACCCTTCTATG	Amplification of *Glyma.* *05G134000*CDS-F
OL13181	AGCATTGAAACTCTGGAAGGGC	Amplification of *Glyma.* *05G134000*CDS-R
OL10628	GCTACACTACACTCCTCAACATTTA	*In situ* hybridization for *GmUFO1*-F
OL10629	CTAAGCATTGAAACTCTGGAAGGGC	*In situ* hybridization for *GmUFO1*-R
OL13180	ATGGAAGGTTTTCACCCTTCTATGT	*In situ* hybridization for *GmUFO2*-F
OL17146	TGTAATACGACTCACTATAGGGCAGCATTAAAAC TCTGGAAGGGAAGTG	*In situ* hybridization for *GmUFO2*-R
OL13183	CTGTACAAGCATATGATGGAAGGTTTTCACCCTT CTATGT	Subcellular localization-F
OL13184	GATGAATTCGAGCTCAGCATTGAAACTCTGGAAG GGC	Subcellular localization-R
OL15707	TGAACATGGGTGGCAATTAC	*GmAP1a*-F for qPCR
OL15708	TGTCAAATGCCATACCAAAG	*GmAP1a*-R for qPCR
OL15715	TCTTGCTCCACCCTTCTCTA	*GmAP2*-F for qPCR
OL15716	CGAGTGGAGGAATGTCATGTT	*GmAP2*-R for qPCR
OL15717	GAGGATAGAGAACACCACCAAC	*GmAP3*-F for qPCR
OL15718	AAACCTTGGCATCGCATAGA	*GmAP3*-R for qPCR
OL15719	CAAAGAGGAAGAATGGGATCCTTA	*GmPI04G*-F for qPCR
OL15720	GGCTGATGTACTCATGCATCT	*GmPI04G*-R for qPCR
OL15725	CAAGAGGATTGAGAACACTACCA	*GmAG*-F for qPCR
OL15726	CTGCATCACAAAGCACAGATAAT	*GmAG*-R for qPCR
OL9129	ATCTTGACTGAGCGTGGTTATTCC	*GmActin*-F for qPCR
OL9130	GCTGGTCCTGGCTGTCTCC	*GmActin*-R for qPCR
OL14387	TTGGGCACGTTCAGCTTGCTCTTCTCA	Knockout test *Glyma.08G088700-R*

Table S2. Protein physical information of 11 *UFO* genes from nine species.

Gene	Motif	Protein length (aa)	Mol. wt. (Da)	PI (pH)	Instability index	GRAVY	Arabidopsis ortholog locus	Arabidopsis locus description
AT1G30950.1	12	442	49,047.32	8.62	56.21	0.001		
Glyma.05G134000.1	13	430	48,378.84	8.71	47.99	-0.024	AT1G30950.1	PROTEIN UNUSUAL FLORAL ORGANS
Glyma.08G088700.1	13	440	49,651.21	8.6	46.36	-0.05	AT1G30950.1	PROTEIN UNUSUAL FLORAL ORGANS
Medtr4g094748.1	13	444	49,946.73	8.98	50.88	-0.017	AT1G30950.1	PROTEIN UNUSUAL FLORAL ORGANS
Gorai.002G139700.1	9	357	39,766.63	8.55	52.26	-0.074	AT1G30950.1	PROTEIN UNUSUAL FLORAL ORGANS
HORVU7Hr1G108970.1	11	438	45,988.19	10.34	60.45	0.224	AT1G30950.1	PROTEIN UNUSUAL FLORAL ORGANS
Os06g45460.1	11	407	42,738.55	9.95	63.51	0.300	AT1G30950.1	PROTEIN UNUSUAL FLORAL ORGANS
Phvul.002G188300.1	13	434	48,843.49	8.74	49.12	0.005	AT1G30950.1	PROTEIN UNUSUAL FLORAL ORGANS
Vigun03g153100.1	13	441	49,447.06	8.74	48.33	-0.005	AT1G30950.1	PROTEIN UNUSUAL FLORAL ORGANS
Zm00001d046950	13	433	45,917.02	9.79	58.42	0.184	AT1G30950.1	PROTEIN UNUSUAL FLORAL ORGANS
Zm00001d036653	13	436	46,192.15	9.27	59.54	0.179	AT1G30950.1	PROTEIN UNUSUAL FLORAL ORGANS

Table S3. The seeds weight per plant of the wild type and *ufo* mutant lines growing in growth chamber under short day conditions.

Genotype	Line1 (g)	Line2 (g)	Line3 (g)	Mean ± SD (g)
Wild type	3.61	3.49	3.66	3.59±0.06
Gmufo1	3.34	3.58	3.87	3.60±0.19
Gmufo2	3.69	3.67	3.54	3.63±0.06
Gmufo1ufo2	3.42	3.68	3.71	3.60±0.11

Table S4. The pedigree of the transgenic lines in this paper.

T0 generation			T1 generation		
Line ID	*UFO1* genotype (Sequence ID)	*UFO2* genotype (Sequence ID)	Line ID	*UFO1* genotype (Sequence ID)	*UFO2* genotype (Sequence ID)
SP2464	*Heterozygous* (SG21525)	*Heterozygous* (SG25372)	SP2464-1 (*ufo1-1*)	*Heterozygous* (SG28123)	WT (SG27198)
			SP2464-2 (*ufo1-2*)	*Heterozygous* (SG24627)	WT (SG24928)
			SP2464-3 (*ufo1-3*)	*Heterozygous* (SG24628)	WT (SG24929)
			SP2464-4 (*ufo1-4*)	*Heterozygous* (SG24629)	WT (SG24930)
			SP2464-5 (*ufo1-5*)	*Heterozygous* (SG24630)	WT (SG24931)
			SP2464-6 (*ufo1-6*)	*Heterozygous* (SG24631)	WT (SG24932)
			SP2464-7 (*ufo1-7*)	*Heterozygous* (SG24632)	WT (SG24933)
			SP2464-9 (*ufo1-9*)	*Heterozygous* (SG24633)	WT (SG24934)
			SP2464-15 (*ufo2-2*)	WT (SG24728)	*Heterozygous* (SG28156)
			SP2464-16 (*ufo1ufo2-17*)	*Homozygous* (SG28118)	*Heterozygous* (SG28157)
SP2465	*Heterozygous* (SG28105)	*Heterozygous* (SG24978)	SP2465-1 (*ufo2-1*)	WT SG24729	*Heterozygous* (SG28153)
			SP2465-2 (*ufo1-15*)	*Heterozygous* (SG28093)	WT (SG24926)
			SP2465-3 (*ufo1ufo2-18*)	*Heterozygous* (SG27192)	*Homozygous* (SG28141)
SP2466	*Heterozygous* (SG24746)	*Heterozygous* (SG24977)	SP2466-1 (*ufo1ufo2-1*)	*Heterozygous* (SG27193)	*Heterozygous* (SG28158)
			SP2466-2 (*ufo1-16*)	*Heterozygous* (SG28120)	WT (SG24925)
			SP2466-3 (*ufo2-3*)	WT (SG24727)	*Heterozygous* (SG28151)
SP2467	WT (SG25354)	*Heterozygous* (SG25370)	SP2467-1 (*ufo2-4*)	WT (SG25351)	*Homozygous* (SG25368)
			SP2467-2 (*ufo2-5*)	WT (SG25352)	*Heterozygous* (SG25369)
			SP2467-3 (*ufo2-6*)	WT (SG25353)	*Homozygous* (SG25371)

Table S5. Comparison of the number of chimeric organs among wild type and *ufo* mutant lines.

Genotype	Sepal-petal chimeric organ (chimeric/total)	Petal-stamen chimeric organ (chimeric/total)
wild type	0/30	0/30
Gmufo1	10/30	1/30
Gmudo2	0/30	0/30
Gmufo1udo2	24/30	3/30

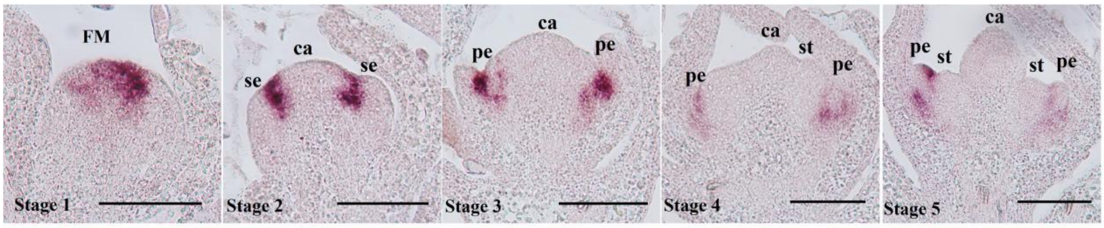

Figure S1. *In situ* hybridization of the *GmUFO2* gene in the soybean flower primordium of different development stages. Bar = 100 μm.

Figure S2. The sequencing results of all T0 and T1 mutant lines.

T0 Generation:

SP2464: *GmUFO1*- SG21525

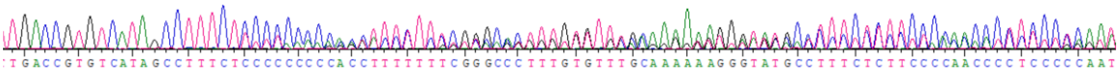

SP2464: *GmUFO2*- SG25372

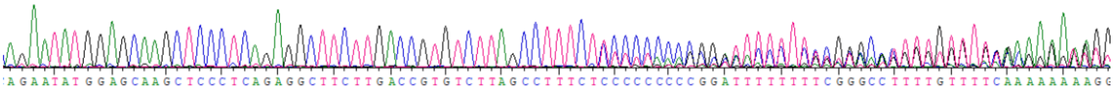

SP2465: *GmUFO1*- SG28105

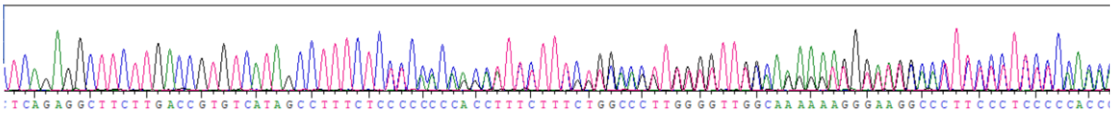

SP2465: *GmUFO2*- SG24978

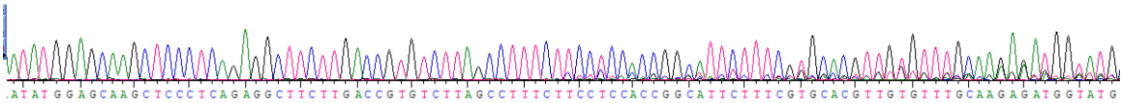

SP2466: *GmUFO1*- SG24746

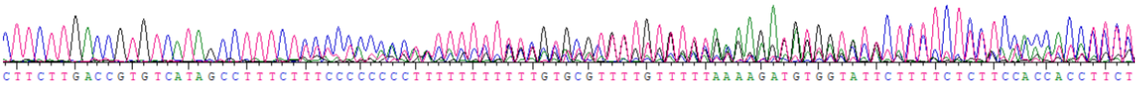

SP2466: *GmUFO2*- SG24977

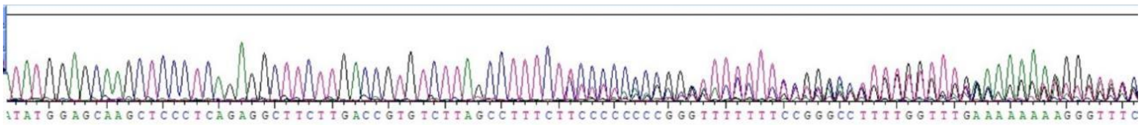

SP2467: *GmUFO1*- SG25354

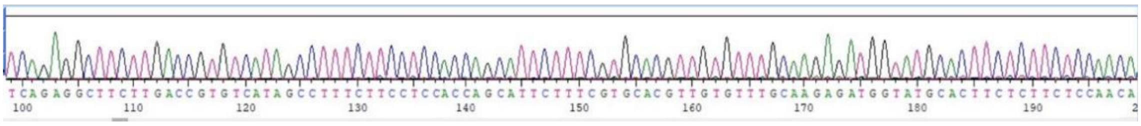

SP2467: *GmUFO2*-SG25370

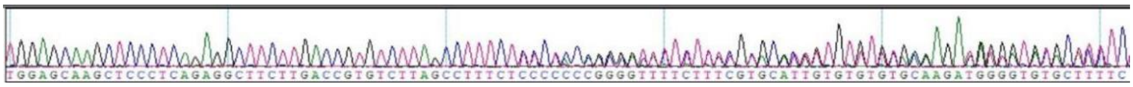

T1 generation:

SP2464-1 (*ufo1-1*): *GmUFO1*-SG28123

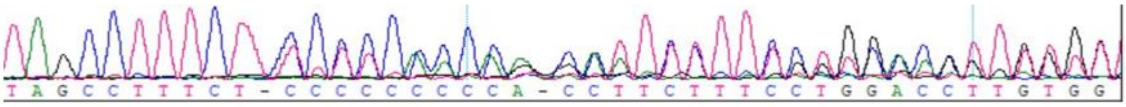

SP2464-1 (*ufo1-1*): *GmUFO2*-SG27198

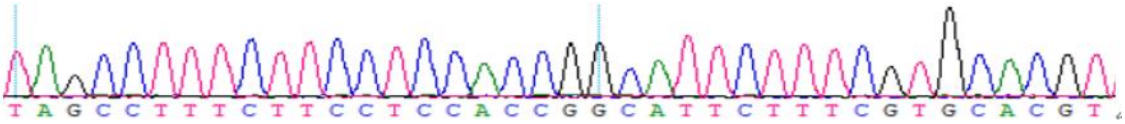

SP2464-2 (*ufo1-2*): *GmUFO1*-SG24627

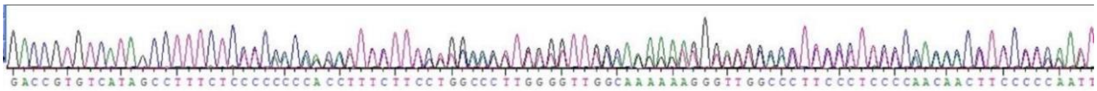

SP2464-2 (*ufo1-2*): *GmUFO2*-SG24928

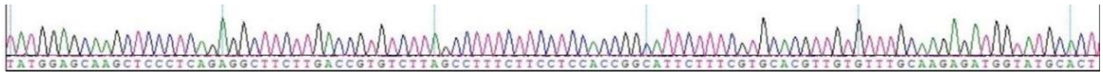

SP2464-3 (*ufo1-3*): *GmUFO1*-SG24628

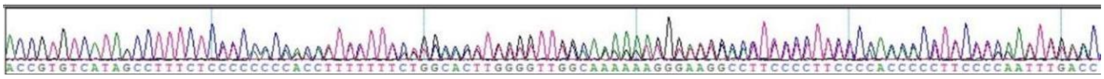

SP2464-3 (*ufo1-3*): *GmUFO2*-SG24929

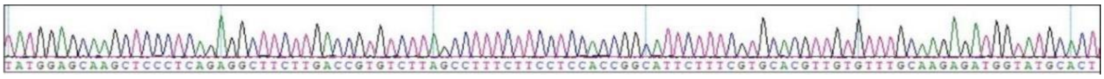

SP2464-4 (*ufo1-4*): *GmUFO1*-SG24629

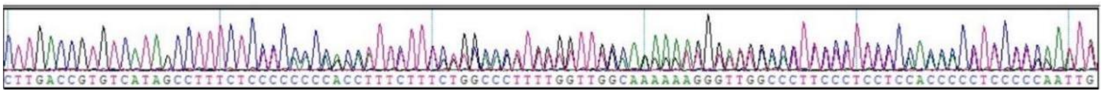

SP2464-4 (*ufo1-4*): *GmUFO2*-SG24930

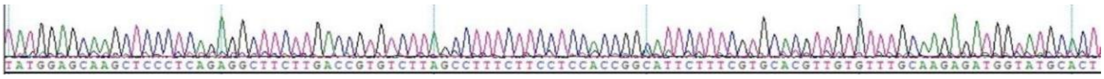

SP2464-5 (*ufo1-5*): *GmUFO1*-SG24630

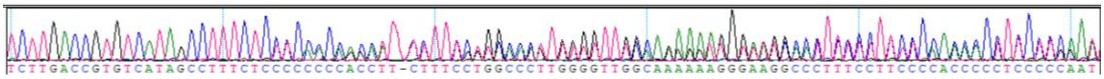

SP2464-5 (*ufo1-5*): *GmUFO2*-SG24931

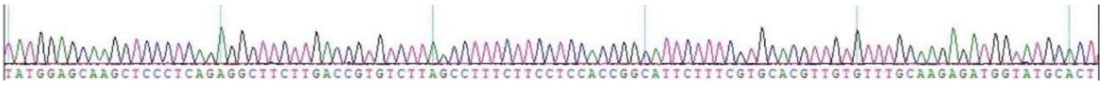

SP2464-6 (*ufo1-6*): *GmUFO1*-SG24631

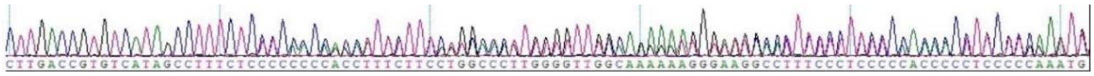

SP2464-6 (*ufo1-6*): *GmUFO2*-SG24932

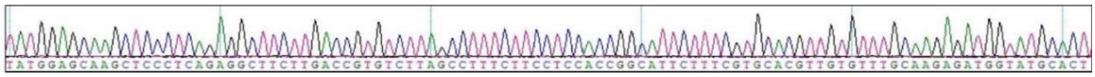

SP2464-7 (*ufo1-7*): *GmUFO1*-SG24632

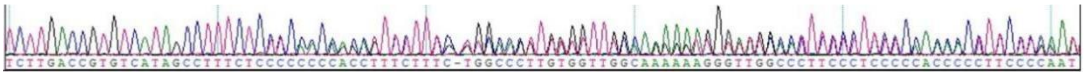

SP2464-7 (*ufo1-7*): *GmUFO2*-SG24933

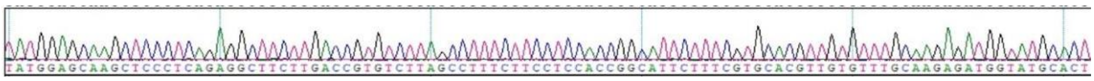

SP2464-9 (*ufo1-9*): *GmUFO1*-SG24633

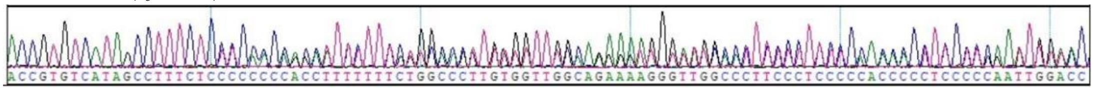

SP2464-9 (*ufo1-9*): *GmUFO2*-SG24934

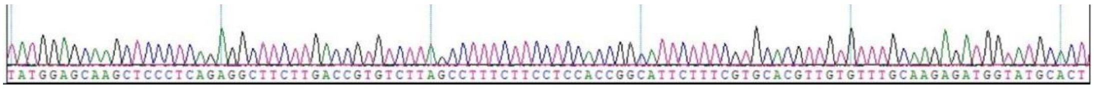

SP2464-15 (*ufo2-2*): *GmUFO1*-SG24728

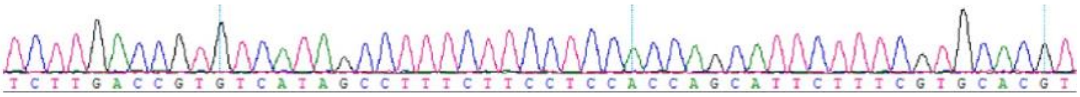

SP2464-15 (*ufo2-2*): *GmUFO2*-SG28156

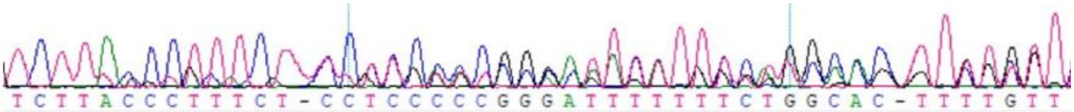

SP2464-16 (*ufo1ufo2-17*): *GmUFO1*-SG28118

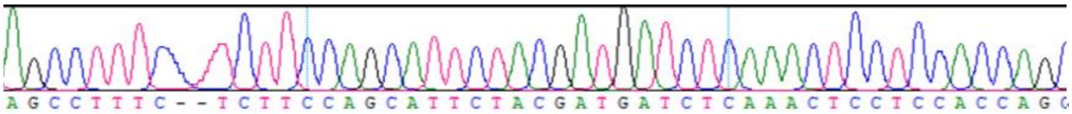

SP2464-16 (*ufo1ufo2-17*): *GmUFO2*-SG28157

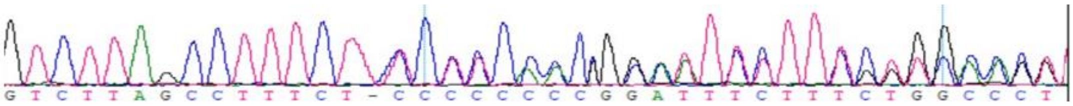

SP2465-1 (*ufo2-1*): *GmUFO1*-SG24729

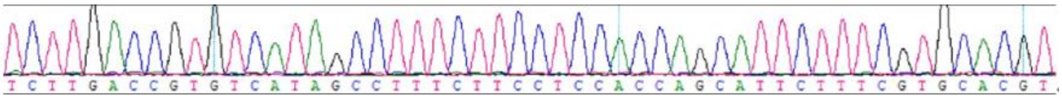

SP2465-1 (*ufo2-1*): *GmUFO2* SG28153

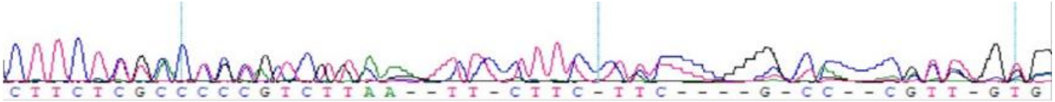

SP2465-2 (*ufo1-15*): *GmUFO1*-SG28093

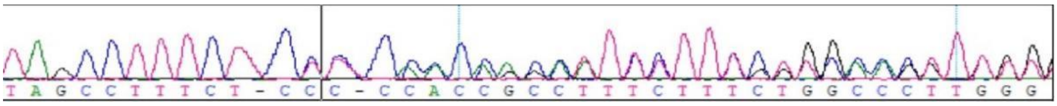

SP2465-2 (*ufo1-15*): *GmUFO*2SG24926

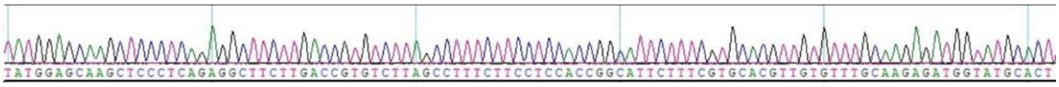

SP2465-3 (*ufo1ufo2-18*): *GmUFO1*-SG27192

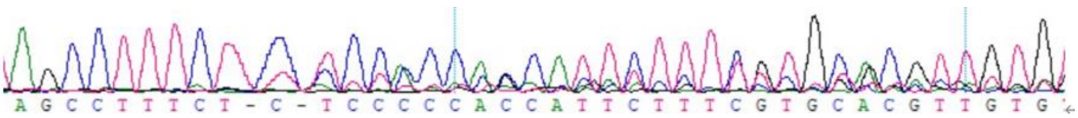

SP2465-3 (*ufo1ufo2-18*): *GmUFO*2SG28141

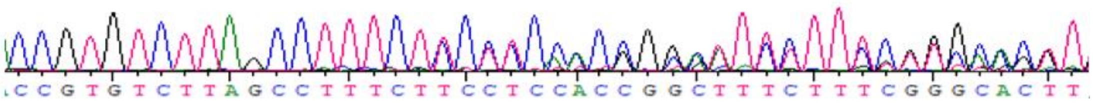

SP2466-1 (*ufo1ufo2-4*): *GmUFO1*-SG27193

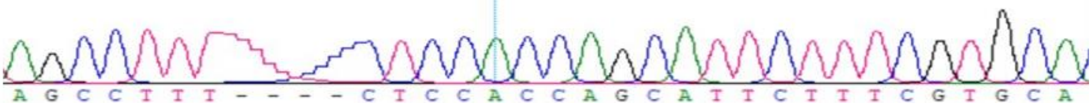

SP2466-1 (*ufo1ufo2-4*): *GmUFO2*SG28158

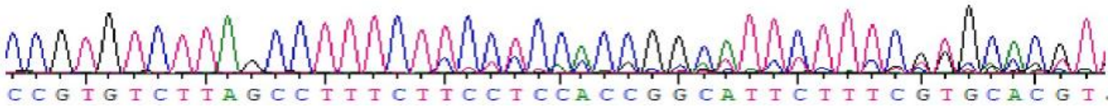

SP2466-2 (*ufo1-16*): *GmUFO1*-SG28120

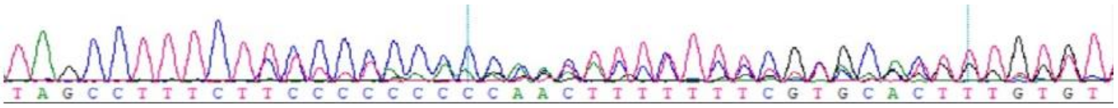

SP2466-2 (*ufo1-16*): *GmUFO2*SG24925

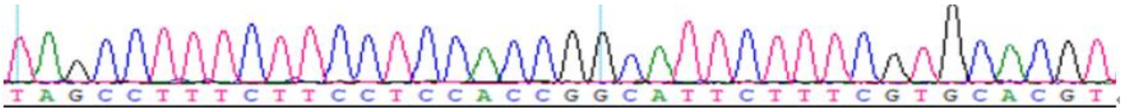

SP2466-3 (*ufo2-3*): *GmUFO1*-SG24727

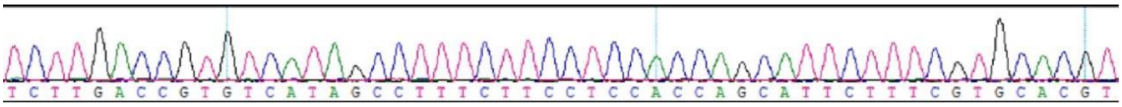

SP2466-3 (*ufo2-3*): *GmUFO2*SG28151

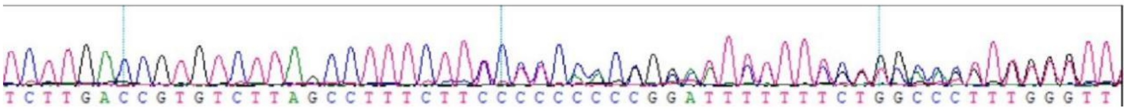

SP2467-1 (*ufo2-4*): *GmUFO1*-SG25351

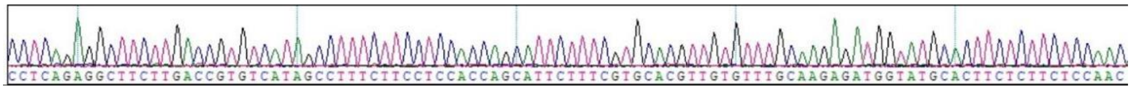

SP2467-1 (*ufo2-4*): *GmUFO2*SG25368

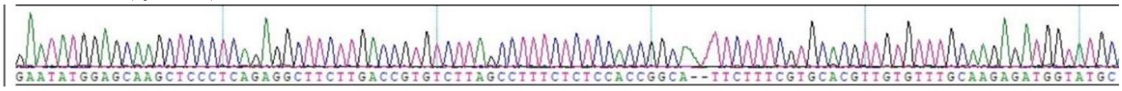

SP2467-2 (*ufo2-5*): *GmUFO1*- SG25352

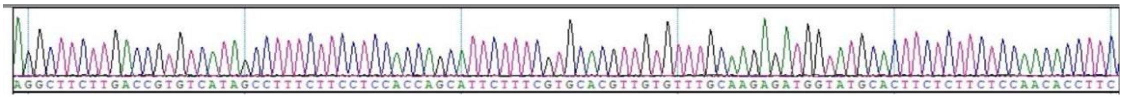

SP2467-2 (*ufo2-5*): *GmUFO2*SG25369

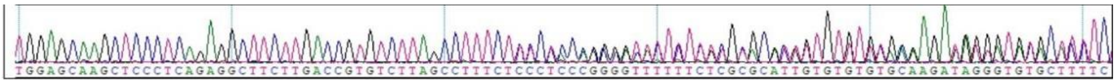

SP2467-3 (*ufo2-6*): *GmUFO1*-SG25353

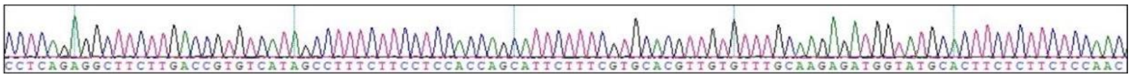

SP2467-3 (*ufo2-6*): *GmUFO2*SG25371

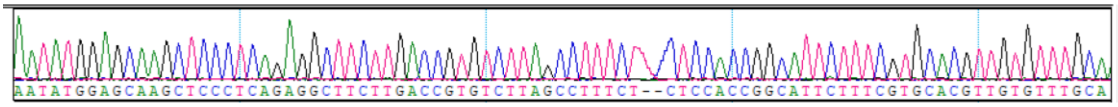

Supplement: Supplementary file 1 [file ijms-24-09662-s001.zip › ijms-2362465-supplementary.pdf]
